# *Camellia nitidissima* Flower Extract Alleviates Stress-Induced Sebaceous Dysfunction by Targeting the 11β-HSD1/PI3K/Akt/mTOR Axis

**DOI:** 10.3390/molecules31183156

**Published:** 2026-09-08

**Authors:** Meng Zhang, Jiayi Fan, Zhenyu Qin, Timson Chen, Zhizhen Li, Ya Chen, Ling Ma, Guang-Li Wang, Jing Wang

**Affiliations:** 1 Key Laboratory of Synthetic and Biological Colloids, Ministry of Education, School of Chemical & Material Engineering, Jiangnan University, Wuxi 214122, China; 6240606040@stu.jiangnan.edu.cn (M.Z.); 6220610019@stu.jiangnan.edu.cn (J.F.); 6240610035@stu.jiangnan.edu.cn (Z.Q.); 2 Adolph Innovation Laboratory, Guangzhou AOGU Cosmetics Co., Ltd., Guangzhou 510520, China; chen2020@126.com (T.C.); lizhizhen@adolph.cn (Z.L.); chenya@haservey.com (Y.C.); maling@adolph.cn (L.M.)

**Keywords:** *Camellia nitidissima* flower extract, sebaceous gland, 11β-HSD1, PI3K/Akt/mTOR, lipogenesis, stress-induced sebaceous dysfunction

## Abstract

Stress-induced sebaceous hyperactivity is a key driver of acne and seborrheic dermatitis; however, research investigating the pathway-specific mechanisms through which stress exerts its effects and the suppression of sebum production via targeting stress signaling remains relatively limited. In this study, we investigated whether *Camellia nitidissima* flower extract (CNF) could counteract stress-induced sebaceous dysfunction and its underlying mechanisms. Using a cortisone-stimulated SZ95 human sebocyte model, we evaluated lipid accumulation, cortisol production, signaling pathway activation, and apoptotic markers. CNF treatment dose-dependently suppressed cortisone-induced lipid production, reducing triglyceride, cholesterol, and free fatty acid levels by up to 35.44%, 38.02%, and 46.39%. Mechanistically, CNF inhibited 11β-HSD1 expression (17.17% reduction) and cortisol secretion (32.84% decrease), thereby blocking local cortisol reactivation. This upstream interception subsequently attenuated PI3K/Akt/mTOR hyperphosphorylation, downregulated lipogenic transcription factors (SREBP-1, PPARγ, LXRα, C/EBP-α) and their target enzymes (FAS, ACC, DGAT). Beyond lipid synthesis inhibition, CNF reversed cortisone-induced apoptosis resistance by reducing the Bcl-2/Bax ratio and suppressing PCNA-mediated hyperproliferation, thereby decreasing sebocyte number. These findings demonstrate that CNF exerts dual oil-control effects: reducing lipid production per cell and reducing lipid-producing cell abundance. Collectively, CNF represents a promising multi-target botanical agent for managing stress-related sebaceous disorders and cosmetic sebum regulation.

## 1. Introduction

Sebaceous gland function homeostasis is the core prerequisite for maintaining intact skin barrier function and cutaneous physiological balance, yet sebocytes are extremely sensitive to systemic psychological and physical stress signals [1]. Chronic stress triggers hyperactivity of the hypothalamic–pituitary–adrenal (HPA) axis [2], elevating circulating glucocorticoid levels and directly driving sebaceous lipogenesis, which acts as a key pathogenic trigger for prevalent inflammatory skin diseases including acne vulgaris and seborrheic dermatitis [3,4]. A critical, often overlooked link in this pathological cascade is the local intrasebaceous conversion of inert cortisone to bioactive cortisol, a reaction dominantly catalyzed by 11β-hydroxysteroid dehydrogenase type 1 (11β-HSD1) [5]. This enzyme serves as a tissue-specific stress signal amplifier within sebocytes [6]: it constructs a localized hypercortisol microenvironment upon overexpression, subsequently fuels abnormal lipid accumulation, and ultimately precipitates sebaceous gland overactivity [7,8]. Beyond its role in skin, 11β-HSD1 is also expressed in liver and adipose tissue, where its inhibition has been validated as a therapeutic strategy for metabolic diseases, supporting the translational relevance of targeting this enzyme in sebaceous dysfunction [9]. However, the specific signaling pathways and downstream targets activated by cortisol to drive sebogenesis remain insufficiently investigated. On the other hand, all existing conventional treatments for acne (e.g., retinoids, oral antibiotics, and hormonal regulators) only suppress downstream lipid overproduction or inflammatory responses, failing to intercept stress signaling at its upstream molecular initiation point-they cannot reverse the 11β-HSD1-mediated local cortisol activation that drives sebaceous dysfunction. Therefore, investigating the precise signaling pathways and molecular targets through which the stress hormone cortisol promotes lipid production, alongside developing innovative active ingredients tailored to counteract stress-induced sebogenesis, represents an attractive and promising research direction.

*Camellia nitidissima*, an endemic rare golden *camellia* species uniquely distributed in the Shiwandashan Mountains of Guangxi, China, is hailed as the “Plant Giant Panda” due to its extremely scarce wild germplasm resources [10]. Its flower extract (CNF) is enriched with high-abundance polyphenols, flavonoids, phenolic acids, and saponins, as well as the characteristic component quercetin-7-O-β-D-glucoside, which distinguishes it from other Camellia species [11]. Existing research has preliminarily verified its robust in vitro antioxidant and anti-inflammatory bioactivities [10,12,13]. However, major research gaps remain in the current literature: no systematic investigation has clarified whether CNF can modulate sebaceous lipid metabolism; whether CNF targets the key stress amplifier 11β-HSD1 to block intracellular cortisol activation; and the complete molecular axis mediating its anti-sebogenic and cytoprotective effects against stress-induced sebocyte damage has never been elucidated. The absence of mechanistic evidence severely restricts the translational application of this rare medicinal plant in oil-control dermatology and skincare formulations, representing a critical research gap this work aims to fill.

In the present study, we aimed to investigate whether CNF could counteract stress-induced sebaceous dysfunction and to elucidate the underlying molecular mechanisms. To this end, we established a cortisone-induced human immortalized sebocyte (SZ95) model to simulate the local stress microenvironment in vitro. Through this model, we systematically evaluated the regulatory effects of CNF on sebaceous lipid synthesis, cortisol production, and key signaling pathways involved in stress-responsive lipogenesis using Western blotting, RT-qPCR, ELISA, and biochemical assays. However, major research gaps remain: no systematic investigation has clarified whether CNF can regulate sebaceous lipid metabolism; whether CNF targets the key stress amplifier 11β-HSD1 to block intracellular cortisol activation; and the complete molecular axis mediating its anti-sebogenic and cytoprotective effects against stress-induced sebocyte damage has never been elucidated. Therefore, this study aims to fill these gaps by investigating whether CNF can counteract cortisone-induced dysfunction in SZ95 sebocytes, elucidating the underlying molecular mechanisms, and evaluating the effects of CNF on sebocyte proliferation and apoptosis.

## 2. Results

### 2.1. Chemical Composition Analysis of CNF

The phytochemical profile of CNF was characterized via HPLC-DAD analysis. Although some high-polarity unlabelled peaks (putatively saccharides or organic acids) were present at the early elution phase, our analysis strategically focused on the polyphenolic and flavonoid fractions responsible for the core bioactivities. Based on comparison of retention times and UV spectra with authenticated reference standards, 10 characteristic bioactive constituents were successfully identified. Rutin and quercetin-7-O-β-D-glucoside were identified as the predominant constituents, while several trace flavonoids were also detected. The phytochemical composition of CNF was indicated in our previous study [14].

### 2.2. Effects of CNF and Cortisone on the Viability of SZ95

**Figure 1:**
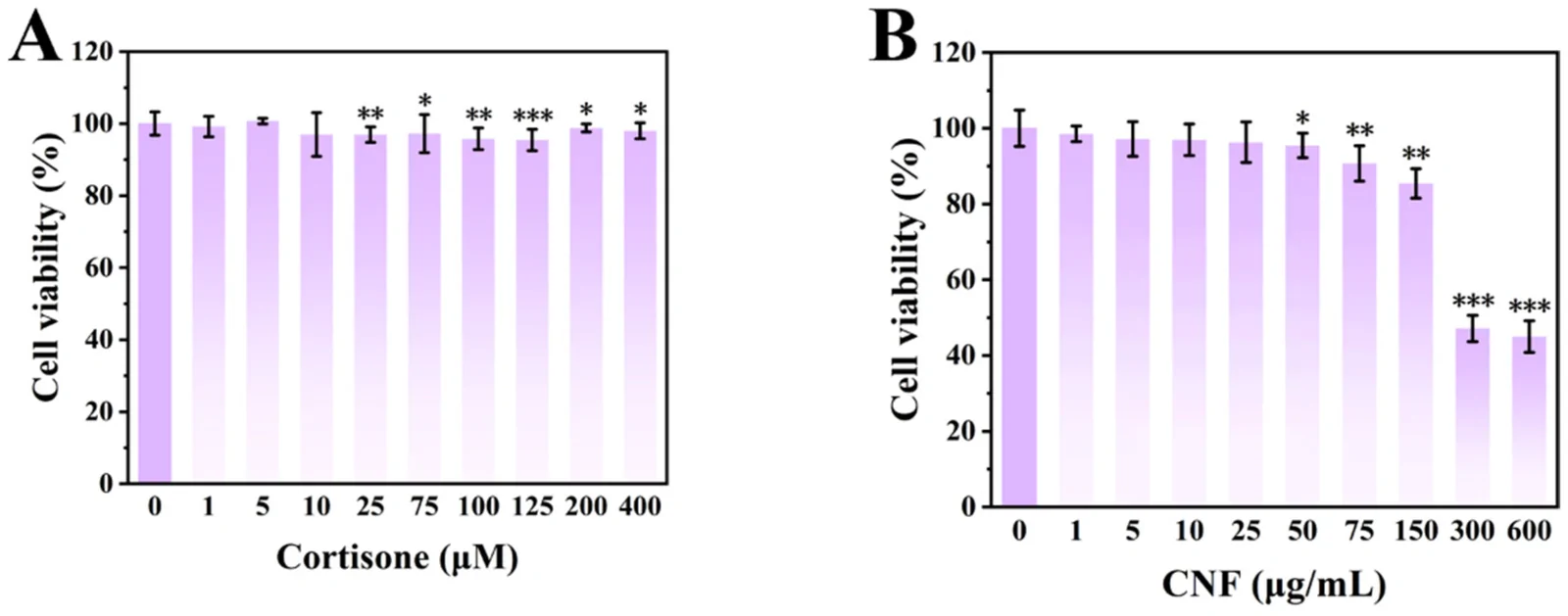
Effects of CNF and cortisone on the activity of SZ95. (**A**) Cytotoxicity of CNF at different concentrations; (**B**) Cytotoxicity of Cortisone at different concentrations (*, ** and *** indicate *p* < 0.05, 0.01 and 0.001 vs. Blank group).

To establish the non-toxic concentration ranges for subsequent experiments, the viability of SZ95 cells treated with CNF and Cortisone was evaluated via MTT assay. As demonstrated in Figure 1, both CNF and Cortisone exhibited high biocompatibility at lower concentrations (1.0–50.0 μg/mL for CNF; 1.0–400.0 μM for Cortisone), maintaining cell viability above 90%. However, cell viability significantly declined to below 80% when CNF concentrations reached the 100.0–600.0 μg/mL range. Based on these toxicological profiles, Cortisone concentrations of 75.0, 100.0, and 125.0 μM and CNF concentrations of 25.0, 50.0, and 75.0 μg/mL were selected for further functional assays. For cortisone, we selected 75, 100, and 125 μM to span the concentration range from submaximal to maximal lipogenic stimulation while maintaining cell viability above 80%. For CNF, we selected 25, 50, and 75 μg/mL to cover the non-toxic concentration range from low to the maximum safe dose (viability > 90%), allowing us to observe dose-dependent effects.

### 2.3. CNF Inhibits Cortisone-Induced Lipid Production

**Figure 2:**
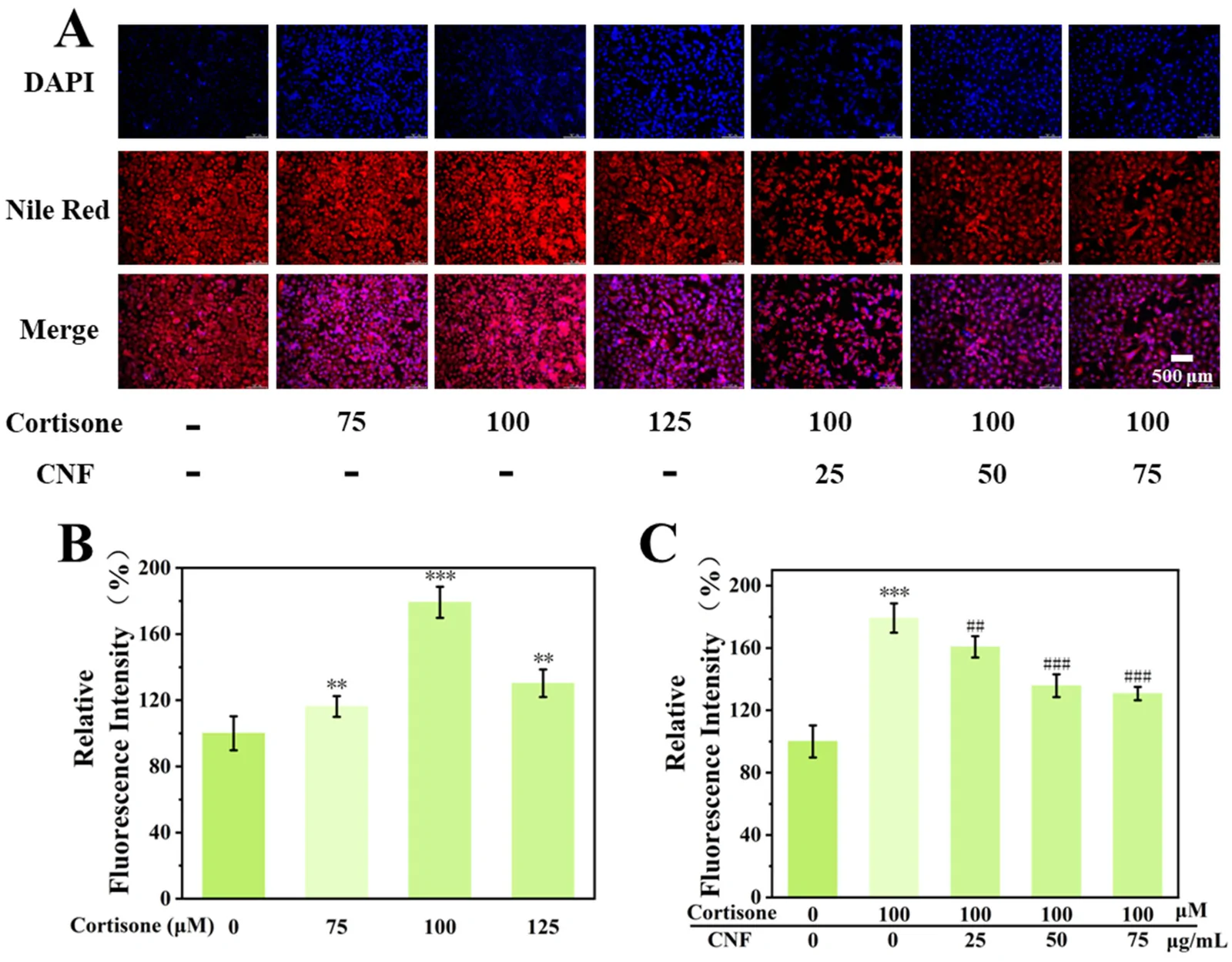
Representative Nile Red staining of intracellular lipids following cortisone treatment. (**A**) Intracellular lipids were stained with Nile Red; (**B**,**C**) Quantitative detection of lipid levels via fluorescence intensity (** and *** indicate *p* < 0.01 and 0.001 vs. Control group; ## and ### indicate *p* < 0.01 and 0.001 vs. Model group).

To simulate a stress microenvironment, SZ95 sebocytes were treated with cortisone, and intracellular lipid production was subsequently evaluated. As shown in Figure 2, Nile Red staining and quantitative analysis revealed that cortisone significantly induced intracellular lipid generation in a dose-dependent manner. At a concentration of 100 μM, cortisone elevated the intracellular lipid content by 79.25%, corresponding to a 1.8-fold increase compared with the blank control group, confirming its potent lipogenic effect. Accordingly, 100 μM cortisone was selected as the stimulant for subsequent experiments to assess the inhibitory effect of CNF on cortisone-induced lipid accumulation. The results demonstrated that CNF at concentrations of 25, 50, and 75 μg/mL suppressed lipid production in a concentration-dependent manner. Notably, at 50 and 75 μg/mL, CNF reduced this elevation to approximately 1.36-fold and 1.31-fold over control, respectively (representing reductions of 24.58% and 27.37% relative to the model group), reversing about 56% of the cortisone-induced lipid accumulation at the highest concentration. These findings suggest that CNF effectively attenuates cortisone-induced total lipid accumulation and holds potential for mitigating excessive sebaceous gland oil production.

### 2.4. Effects of CNF on Triglyceride, Cholesterol, and Fatty Acid Levels

**Figure 3:**
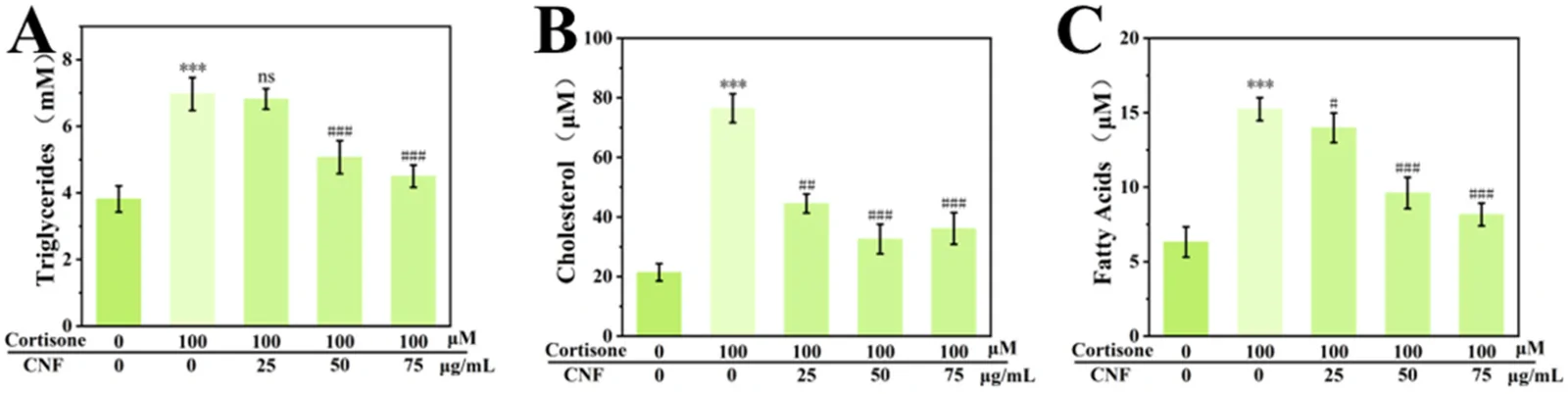
CNF inhibits cortisone-induced intracellular lipid biosynthesis and cortisol production in SZ95 sebocytes (**A**) Triglycerides levels; (**B**) Cholesterol levels; (**C**) Free fatty acid levels (*** indicates *p* < 0.001 vs. Control group; #, ##, and ### indicate *p* < 0.05, 0.01 and 0.001 vs. Model group; ns indicates not significant).

To further elucidate the inhibitory effect of CNF on sebaceous lipid biosynthesis, we quantified the levels of key lipid components, including triglycerides, total cholesterol, and free fatty acids (Figure 3). The results demonstrated that cortisone stimulation (100 μM) significantly promoted the production of all three lipid species compared with the blank control group. In contrast, CNF treatment markedly reversed these biochemical alterations. Specifically, as shown in Figure 3, CNF reduced the accumulation of triglycerides, cholesterol, and free fatty acids in a concentration-dependent manner. At a concentration of 50 μg/mL, CNF decreased the levels of triglycerides, total cholesterol, and free fatty acids by 27.26%, 35.91%, and 36.88%, respectively, relative to the model group. When the concentration was increased to 75 μg/mL, the reductions reached 35.44%, 38.02%, and 46.39%, respectively. Collectively, these findings demonstrate that CNF effectively suppresses cortisone-induced excessive lipogenesis in sebocytes, highlighting its potent lipid-regulatory capacity.

### 2.5. CNF Suppresses Cortisone-Induced 11β-HSD1 and Cortisol Expression

**Figure 4:**
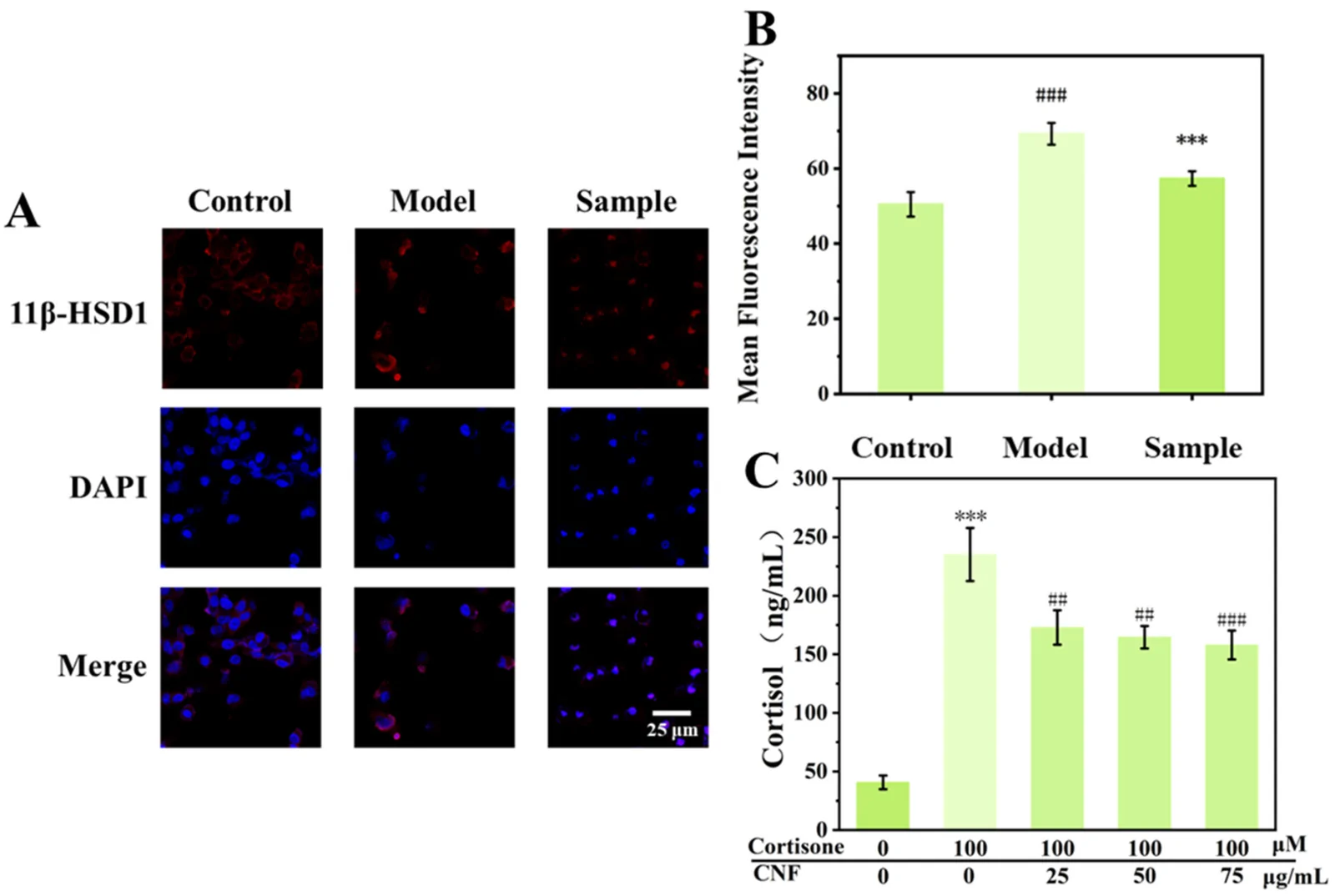
CNF inhibits cortisone-triggered upregulation of 11β-HSD1 protein and cortisol overproduction in SZ95 cells under different treatment conditions: Control (untreated), Model (treated by 100 mM of Cortisone), and Sample (treated by the mixture of 100 μM of cortisone and 75 μg/mL of CNF); (**A**) Representative immunofluorescence images; (**B**) Quantitative analysis of the mean fluorescence intensity of 11β-HSD1, (**C**) ELISA quantification of intracellular cortisol levels (*** indicates *p* < 0.001 vs. Control group; ## and ### indicate *p* < 0.01 and 0.001 vs. Model group).

To elucidate the upstream regulatory mechanisms underlying the oil-control effect mediated by CNF, we examined the expression of 11β-HSD1, a key enzyme that governs local glucocorticoid activity by converting inactive cortisone into bioactive cortisol. On this basis, SZ95 cells were treated with cortisone alone or in combination with CNF, and the effects of CNF on cortisone-induced 11β-HSD1 expression and cortisol production were evaluated via ELISA and immunofluorescence staining. As shown in Figure 4, compared with the cortisone-alone group, treatment with 50 μg/mL CNF reduced the immunofluorescence intensity of 11β-HSD1 by 17.17% and decreased cortisol levels by 32.84%. Collectively, these findings indicate that CNF significantly attenuates 11β-HSD1 signaling, thereby limiting the conversion of inactive cortisone to active cortisol and effectively counteracting the local stress response at its source.

### 2.6. CNF Inhibits Lipid Synthesis via the PI3K/Akt/mTOR Pathway

**Figure 5:**
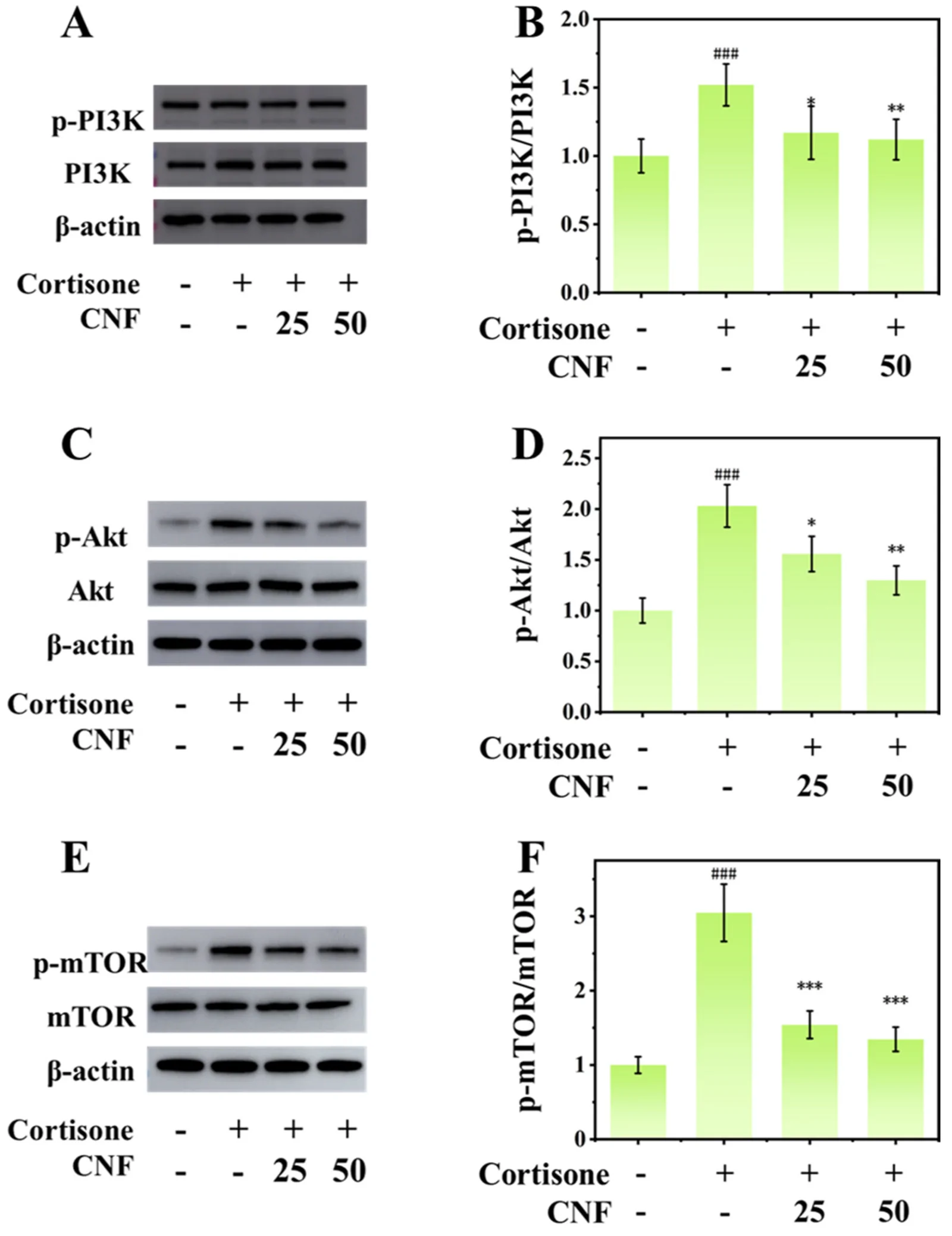
CNF inhibits the cortisone-induced activation of the PI3K/Akt/mTOR signaling pathway in SZ95 cells. (**A**) Expression of p-PI3K and total PI3K; (**B**) Quantitative analysis of p-PI3K; (**C**) Expression of p-Akt and total Akt; (**D**) Quantitative analysis of p-Akt; (**E**) Expression of p-mTOR and total mTOR; (**F**) Quantitative analysis of p-mTOR (### indicates *p* < 0.001 vs. Control group; *, ** and *** indicate *p* < 0.05, 0.01 and 0.001 vs. Model group).

To further elucidate the molecular mechanism by which CNF alleviates cortisone-induced excessive sebaceous lipogenesis, we examined the activity of the PI3K/Akt/mTOR signaling pathway and its downstream lipogenic transcriptional regulatory network. As shown in Figure 5, Western blot analysis revealed that cortisone stimulation significantly increased the phosphorylation levels of PI3K, Akt, and mTOR in SZ95 cells, indicating aberrant hyperactivation of this signaling cascade. CNF treatment markedly reduced the phosphorylation levels of these proteins in a concentration-dependent manner, without affecting their total protein expression.

**Figure 6:**
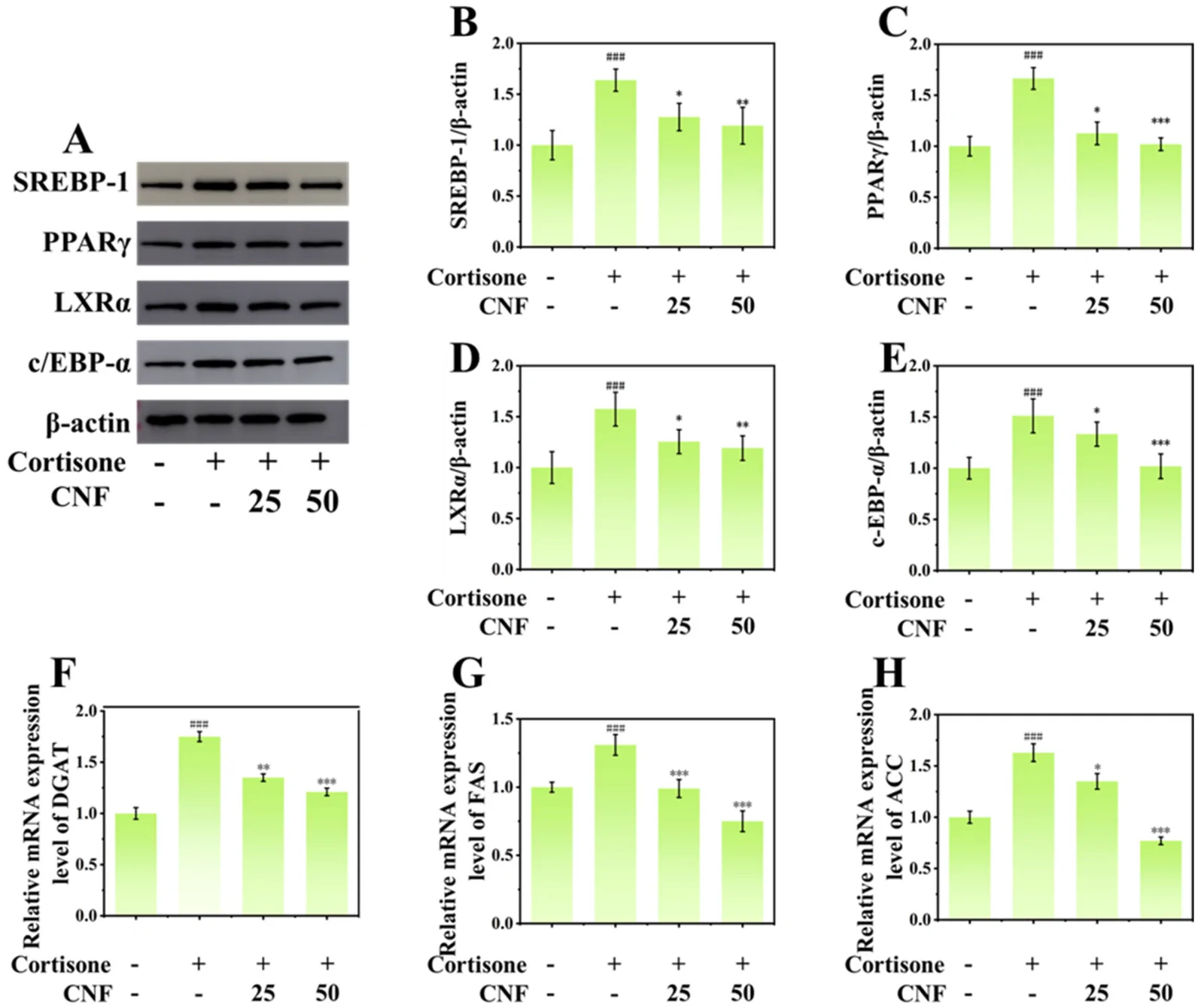
CNF downregulates cortisone-induced expression of lipogenic transcription factors and their downstream target enzymes in SZ95 cells. (**A**) Western blot bands; (**B**) SREBP-1/β-actin; (**C**) PPARγ/β-actin; (**D**) LXRα/β-actin; (**E**) c-EBP-α/β-actin; (**F**) DGAT; (**G**) FAS; (**H**) ACC (### indicates *p* < 0.001 vs. Control group; *, ** and *** indicate *p* < 0.05, 0.01 and 0.001 vs. Model group).

We next investigated the expression of downstream lipogenic transcription factors and synthases using RT-qPCR and Western blotting. As presented in Figure 6, cortisone significantly upregulated the protein expression levels of SREBP-1, PPARγ, LXRα, and C/EBP-α. Consistently, the protein levels of their downstream target enzymes—FAS, ACC, and DGAT—were also elevated accordingly. Notably, CNF intervention effectively reversed the expression of these transcription factors and synthases, restoring them to levels comparable to those in the control group. Collectively, these results demonstrate that CNF suppresses the activation of the PI3K/Akt/mTOR signaling pathway, thereby shutting down the lipogenic program at the transcriptional level.

### 2.7. CNF Attenuates Cortisone-Induced Sebocyte Apoptosis

**Figure 7:**
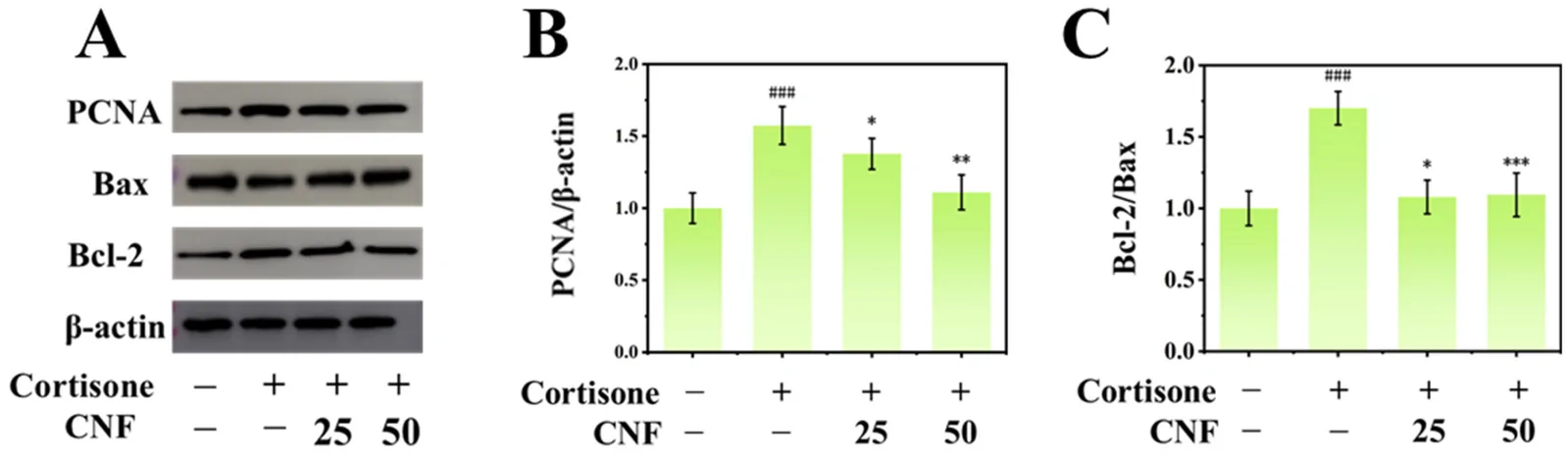
CNF reverses cortisone-induced apoptosis resistance and suppresses excessive proliferation in SZ95 cells (**A**) Western blot bands; (**B**) Quantitative analysis of the Bcl-2/Bax ratio; (**C**) Quantitative analysis of PCNA protein expression. (### indicates *p* < 0.001 vs. Control group; *, ** and *** indicate *p* < 0.05, 0.01 and 0.001 vs. Model group).

We evaluated the effects of CNF on cortisone-induced apoptosis and proliferation in SZ95 cells. As shown in Figure 7, Western blot analysis revealed that cortisone treatment increased the expression of the anti-apoptotic protein Bcl-2, decreased the expression of the pro-apoptotic protein Bax, and consequently elevated the Bcl-2/Bax ratio, indicating that cortisone induced an apoptosis-resistant state in sebocytes. Upon CNF treatment, Bcl-2 expression was markedly downregulated, Bax expression was upregulated, and the Bcl-2/Bax ratio was significantly reduced, demonstrating that CNF reversed the cortisone-induced apoptosis resistance and shifted the cells toward a pro-apoptotic state. In addition, cortisone stimulation significantly upregulated the expression of the proliferation marker PCNA, an effect that was effectively reversed by CNF treatment. Collectively, these results demonstrate that CNF not only inhibits cortisone-induced excessive proliferation of sebocytes but also reverses apoptosis resistance by modulating the Bcl-2/Bax balance, thereby achieving long-term sebum control by reducing sebocyte number.

## 3. Discussion

Stress-induced sebaceous gland hyperactivity is a hallmark of various dermatological conditions; however, the precise molecular mechanisms linking systemic stress to local lipid dysregulation remain incompletely understood. In the present study, we systematically investigated the anti-sebogenic and cytoprotective properties of CNF using a cortisone-induced SZ95 sebocyte model. Our findings demonstrate that CNF functions as a multi-target modulator that alleviates stress-induced sebaceous dysfunction by inhibiting the 11β-HSD1/PI3K/Akt/mTOR signaling axis. This mechanistic dissection not only deepens our understanding of stress–sebum crosstalk but also establishes CNF as a promising botanical candidate with both therapeutic and cosmetic potential.

A central finding of this study is the identification of 11β-HSD1 as a key upstream target of CNF. Within the sebaceous gland, 11β-HSD1 catalyzes the local conversion of inactive cortisone to active cortisol, functioning as a local amplifier of systemic stress signals. This enzyme has been increasingly recognized as a critical node in peripheral glucocorticoid metabolism, with emerging evidence linking its dysregulation to metabolic syndrome, obesity, and cutaneous disorders. In the context of sebaceous biology, previous studies have shown that 11β-HSD1 expression correlates positively with sebum secretion rates in patients with acne vulgaris, suggesting that local cortisol reactivation may serve as a bridge between systemic stress and dermatological manifestations [15]. Our findings verified that cortisone stimulation markedly upregulated the expression of 11β-HSD1 and cortisol production in SZ95 cells, while CNF intervention efficiently reversed this promoting effect. This finding is particularly noteworthy because it positions CNF as an intervention that intercepts stress signaling at its entry point into the sebocyte, rather than merely counteracting downstream effects. To our knowledge, this is the first report that a botanical extract from *C. nitidissima* can suppress 11β-HSD1 expression in human sebocytes, providing a mechanistic rationale for its use in stress-related sebaceous disorders. The phytochemical basis underlying this inhibitory effect warrants further investigation.

Building upon these observations, we identified the PI3K/Akt/mTOR axis as the central molecular switch linking 11β-HSD1-mediated stress to downstream lipogenic gene expression. The PI3K/Akt/mTOR pathway is a well-established central regulator of cell growth, survival, and metabolism, and its hyperactivation has been implicated in various hyperproliferative and metabolic disorders [15]. In sebocytes, this pathway mediates the lipogenic effects of androgens, insulin, and growth factors, positioning it as a convergent node for diverse pro-sebogenic stimuli. Our Western blot data confirmed that cortisone triggers hyperactivation of this signaling cascade, as evidenced by increased phosphorylation levels of PI3K, Akt, and mTOR [16]. CNF treatment markedly attenuated these phosphorylation events in a concentration-dependent manner, indicating a robust negative regulatory effect [17]. These transcription factors orchestrate a coordinated lipogenic program [18]. This finding aligns with previous reports that the PI3K/Akt/mTOR pathway is a critical regulator of lipogenesis in sebocytes and that its pharmacological inhibition reduces sebum production. Notably, our data further revealed that CNF downregulated the expression of key lipogenic transcription factors—SREBP-1, PPARγ, LXRα, and C/EBP-α [19,20,21]—as well as their downstream target enzymes FAS, ACC, and DGAT [22,23]. These transcription factors orchestrate a coordinated lipogenic program: SREBP-1 drives the expression of fatty acid synthases, PPARγ promotes adipogenic differentiation, LXRα senses lipid intermediates and amplifies lipogenic signals, and C/EBP-α cooperates with PPARγ to potentiate transcriptional output [16,24,25]. The concurrent downregulation of all four transcription factors suggests that CNF exerts broad and coordinated suppression of the lipogenic transcriptional network, rather than targeting a single node. These results indicate that CNF suppresses the lipogenic program at the transcriptional level, a mechanism that distinguishes it from simple lipid-absorbing agents that merely sequester surface sebum without affecting its biosynthesis.

In addition to its regulatory effects on lipid biosynthesis, CNF also exerted a profound influence on the survival dynamics of sebocytes. The Bcl-2 family of proteins plays a critical role in the intrinsic apoptotic pathway, which is governed by the balance between anti-apoptotic members (Bcl-2) and pro-apoptotic members (Bax) [26]. An elevated Bcl-2/Bax ratio favors cell survival. In the present study, cortisone stimulation led to a marked increase in the Bcl-2/Bax ratio in SZ95 sebocytes, indicating the establishment of an apoptosis-resistant state. Although glucocorticoids typically upregulate pro-apoptotic factors and downregulate anti-apoptotic Bcl-2 family members in lymphoid and certain epithelial cells, the contrasting response observed in sebocytes may reflect their unique adaptive characteristics under stress conditions [27]. This stress-induced survival state, characterized by an elevated Bcl-2/Bax ratio and enhanced proliferation, may initially represent an adaptive response under acute stress. However, under chronic glucocorticoid exposure, this sustained anti-apoptotic and pro-proliferative state becomes maladaptive, leading to sebocyte accumulation and sebaceous gland hyperplasia—a hallmark of acne-prone skin. This provides a mechanistic link between chronic stress-induced apoptosis resistance and acne pathogenesis [28]. The results of cortisone stimulation showed significantly upregulated expression of the proliferation marker PCNA and apoptosis resistance, while the same concentration appeared cytotoxic in the MTT assay. This apparent discrepancy can be understood by considering the unique biological characteristics of sebocytes, which undergo holocrine secretion—a process in which mature sebocytes accumulate large amounts of lipids, undergo terminal differentiation, and then rupture to release sebum [29,30]. When 100 μM cortisone drives excessive lipogenesis, it accelerates the terminal differentiation and holocrine rupture of mature, lipid-laden sebocytes. This physiological depletion of mature cells results in a modest reduction in the MTT signal across the entire cell population. Importantly, this reflects metabolic exhaustion caused by hyperactive lipid synthesis rather than classical chemical cytotoxicity, which is consistent with our observation that cell viability remained above 90% in the MTT assay. Concurrently, to compensate for the loss of differentiated sebocytes and maintain the lipid-producing cell pool, the remaining basal/surviving cells activate a compensatory program [31]. Western blot analysis, which primarily detects protein expression in these adherent viable cells, revealed upregulation of PCNA and elevation of the Bcl-2/Bax ratio—indicating enhanced proliferation and anti-apoptotic signaling. Together, these two readouts provide complementary evidence for the pathological dynamics of stress-induced sebaceous gland hyperactivity—a condition characterized by both excessive lipid output and compensatory cellular hyperplasia. Therefore, the modest reduction observed in MTT signal reflects the physiological depletion of mature sebocytes undergoing holocrine rupture due to excessive lipogenesis, rather than classical chemical cytotoxicity—consistent with cell viability remaining above 90%. Meanwhile, the upregulation of PCNA and elevation of the Bcl-2/Bax ratio in the remaining basal cells represent a compensatory proliferation and survival program to maintain the sebocyte pool. To conclude, these two observations are not contradictory but rather complementary, together depicting a state of stress-induced sebaceous hyperactivity characterized by both excessive lipid output and compensatory cellular hyperplasia. However, under chronic stress, this survival program may be pathologically co-opted, leading to sebocyte accumulation, glandular hypertrophy, and sustained sebum overproduction—a phenotype reminiscent of the “stress-induced survival” phenomenon described in certain cancer cells. Notably, CNF treatment effectively reversed this apoptosis-resistant phenotype by downregulating Bcl-2 expression, upregulating Bax expression, and consequently reducing the Bcl-2/Bax ratio [32]. Furthermore, CNF suppressed the cortisone-induced upregulation of PCNA, a well-established marker of proliferation that plays an essential role in DNA replication and cell cycle progression. Collectively, these findings indicate that CNF exerts a dual beneficial effect: it not only attenuates lipid synthesis per cell by suppressing the lipogenic transcriptional network, but also reduces the total number of lipid-producing cells by inhibiting proliferation and reinstating apoptotic susceptibility, thereby providing a cellular basis for long-term sebum control [33].

This study has several limitations. First, our experiments were conducted exclusively in an immortalized human sebocyte cell line (SZ95) rather than in primary sebocyte cultures, ex vivo skin explants, or in vivo animal models. Although SZ95 cells are widely accepted as a reliable in vitro model and have been extensively used in sebaceous gland research, they may not fully recapitulate the complex microenvironment of native sebaceous glands, including interactions with surrounding keratinocytes, fibroblasts, immune cells, and the neural and vascular components that constitute the pilosebaceous unit. In addition, our in vitro model cannot fully capture the neuro–immuno–endocrine network of the skin, which is known to produce glucocorticoids locally in an organized manner via a peripheral equivalent of the HPA axis [34]. Future studies employing ex vivo skin explants, three-dimensional sebocyte organoids, or in vivo animal models will be valuable for validating our findings under more physiologically relevant conditions.

**Figure 8:**
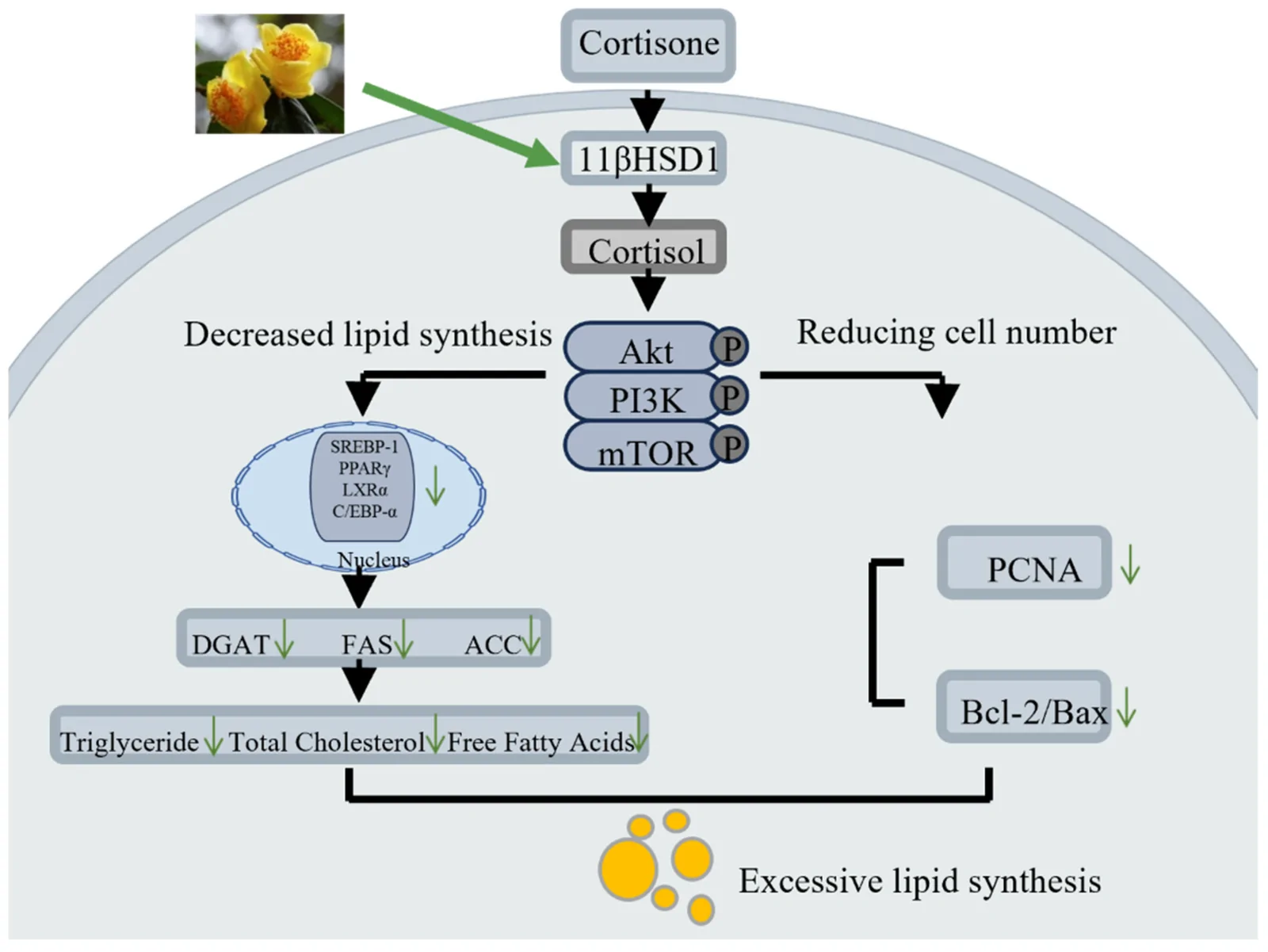
Proposed mechanism by which CNF mitigates cortisone-induced sebaceous gland dysfunction.

As summarized in Figure 8, this study demonstrates that CNF effectively alleviates stress-induced sebaceous gland dysfunction. Mechanistically, CNF inhibits the expression of 11β-HSD1, thereby blocking the local conversion of cortisone to active cortisol and suppressing the aberrant activation of the PI3K/Akt/mTOR signaling pathway. This multi-target regulatory effect not only downregulates the expression of lipogenic transcription factors such as SREBP-1 and PPARγ, as well as their downstream target enzymes FAS, ACC, and DGAT, thereby reducing lipid synthesis, but also reverses the cortisone-induced apoptosis-resistant state, reduces the Bcl-2/Bax ratio, and suppresses the expression of the proliferation marker PCNA, thereby reducing sebocyte number. Consequently, CNF exerts its sebum-control effects not only by reducing lipid production per cell but also by reducing the number of lipid-producing cells, achieving long-term efficacy. This work provides the first mechanistic evidence that CNF acts as a multi-target botanical agent against stress-induced sebaceous overactivity, filling a critical gap in the application of this rare plant in dermatological and cosmetic oil-control strategies. Collectively, CNF, as a multi-functional natural active ingredient derived from the rare plant *Camellia nitidissima*, holds broad application prospects in both the clinical treatment of stress-related sebaceous disorders and cosmetic sebum regulation.

## 4. Materials and Methods

### 4.1. Extraction, Purification and Phytochemical Characterization of CNF

#### 4.1.1. Extraction of CNF

*Camellia nitidissima* flowers (CNF) were purchased from Dongxing Zhengda Trading Co., Ltd. (Dongxing, China), a licensed supplier with legal collection authorization. The species was authenticated by the supplier and confirmed in our laboratory. A voucher specimen has been deposited at the Guangxi Museum of Natural Resources (Nanning, China). *Camellia nitidissima* is a seasonal plant with a flowering period from January to April, peaking from late February to mid-March. The flowers used in this study were harvested at the full-bloom stage to ensure consistency of bioactive constituents, as previous studies have demonstrated that this stage exhibits the most comprehensive profile of polyphenols and flavonoids. The dried flowers were ground and extracted under optimized conditions to obtain the CNF extract. Fresh CNF was freeze-dried and mechanically pulverized into a fine powder. The powder was extracted with 55% ethanol at a solid-to-liquid mass ratio of 1:20 in a 70 °C water bath for 3 h, and the extraction was repeated twice. The combined extracts were filtered through a 0.22 μm filter membrane to remove insoluble residues. The filtrate was concentrated under reduced pressure using a rotary evaporator to remove ethanol, yielding a viscous aqueous solution. The concentrated solution was further filtered and then spray-dried to obtain the final *Camellia nitidissima* flower extract (CNF) powder, which was stored in a desiccator at room temperature in the dark until further use.

#### 4.1.2. Phytochemical Characterization of CNF

The phytochemical profile of CNF was characterized using high-performance liquid chromatography coupled with a diode-array detector (HPLC-DAD) (Waters Corporation, Milford, MA, USA). Chromatographic separation was performed on a Phenomenex C18 column (5 μm, 4.6 × 250 mm) via gradient elution with 0.1% aqueous formic acid (mobile phase A) and acetonitrile (mobile phase B) at a constant flow rate of 0.6 mL/min over 90 min. UV detection was monitored at wavelengths of 200, 254, and 350 nm. Major constituents were tentatively identified by comparing their retention times and UV absorption spectra with those of authentic reference standards.

### 4.2. Cell Culture

The immortalized human sebaceous gland cell line SZ95, kindly provided by Prof. Christos C. Zouboulis (Brandenburg Medical School Theodor Fontane, Germany), was cultured in DMEM supplemented with 10% fetal bovine serum (Gibco, Carlsbad, CA, USA), 100 U/mL penicillin, and 100 μg/mL streptomycin at 37 °C in a humidified atmosphere containing 5% CO_2_. For subculture, cells were detached using trypsin–EDTA (0.25%, Gibco) and propagated in fresh complete medium under the same conditions.

### 4.3. MTT Assay for SZ95 Cell Proliferation

The cytotoxicity of cortisone and CNF was evaluated using the 3-(4,5-dimethylthiazol-2-yl)-2,5-diphenyltetrazolium bromide (MTT) assay. Stock solutions of CNF (1.0 mg/mL, prepared by dissolving 10 mg of CNF powder in 10 mL of DMEM) and cortisone (138.8 mM, prepared by dissolving 100 mg of cortisone in 2.0 mL of DMSO) were serially diluted with DMEM to obtain the desired working concentrations. The final DMSO concentration in the cortisone-treated groups was 0.07% (*v*/*v*), which is well below the widely accepted threshold of 0.1% (*v*/*v*) reported to affect cell viability or metabolic activity in mammalian cell lines. SZ95 cells were seeded into 96-well plates at a density of 1 × 10^5^ cells/mL (100 μL per well). After 24 h of culture, the cells were treated with 100 μL of various concentrations of CNF or cortisone, while the control group received an equal volume of DMEM (sextuplicate wells for each condition). Following 48 h of incubation, 100 μL of MTT solution (0.5 mg/mL; Sigma-Aldrich, St. Louis, MO, USA) was added to each well and incubated for an additional 4 h. The supernatant was then carefully removed, and 100 μL of DMSO (Sinopharm Chemical Reagent Co., Ltd., Beijing, China) was added to dissolve the formazan crystals. The absorbance was measured at 490 nm using a microplate reader (Tecan Group Ltd., Maennedorf, Switzerland). Cell viability was calculated according to the following Formula (1):

(1) $$\mathrm{Cell}\;\mathrm{viability}\;(\%)\;=\;\frac{A_{1}}{A_{2}}\;\times \;100\%$$

where A_1_ and A_2_ represent the average OD values of the experimental and the blank groups at 490 nm, respectively.

### 4.4. Nile Red Staining and Lipid Content Measurement

The selection of 100 μM cortisone was based on the dose–response curve, where 100 μM represented the lowest concentration that produced a significant and reproducible lipogenic effect without causing excessive cytotoxicity (cell viability >80%). The highest concentration of CNF (75 μg/mL) was selected as it was the maximum concentration that maintained cell viability above 90% and was therefore considered the optimal non-toxic dose for functional assays. To evaluate intracellular lipid accumulation, SZ95 cells were treated with either 100 μL of cortisone (100.0 μM) alone or a combination of cortisone and various concentrations of CNF for 48 h. Subsequently, lipid content was assessed using Nile Red staining. Briefly, cells were incubated with 100 μL of DAPI solution (Beyotime Inc., Shanghai, China) for 10 min at room temperature to stain the nuclei. After removing the DAPI solution, 100 μL of Nile Red solution (10 μg/mL in PBS) was added to each well and incubated at 37 °C in the dark for 20 min. Stained cells were imaged using a fluorescence microscope (Leica Stellaris 5, Germany), and the fluorescence intensity of Nile Red was measured at excitation and emission wavelengths of 485 nm and 565 nm, respectively. Fluorescence intensity was quantified using ImageJ 1.47v software to evaluate intracellular lipid accumulation.

### 4.5. Immunofluorescence Staining for 11β-HSD1

SZ95 cells were seeded into laser confocal dishes at a density of 7 × 10^4^ cells/mL (1 mL per dish) and treated with either cortisone (100 μM) alone or a combination of cortisone (100 μM) and CNF (75 μg/mL). After 24 h of incubation, the cells were washed with PBS and fixed with immunostaining fixative for 15 min. Following washing, the cells were blocked with immunostaining blocking buffer for 1 h at room temperature. Thereafter, the cells were incubated with primary antibody against 11β-HSD1 overnight at 4 °C, followed by incubation with the corresponding secondary antibody for 2 h. After washing, the nuclei were stained with an antifade mounting medium containing DAPI for 10 min. Images were captured using a laser scanning confocal microscope (Leica Microsystems, Wetzlar, Germany). All reagents used for immunofluorescence were obtained from Beyotime Biotechnology Co., Ltd. (Shanghai, China). Fluorescence intensity was quantified using ImageJ software to evaluate 11β-HSD1 expression levels.

### 4.6. Determination of Cortisone-Induced Cortisol Production

To determine cortisone-induced cortisol production, SZ95 cells were seeded into 6-well plates at a density of 1.5 × 10^5^ cells/mL (2 mL per well) and allowed to adhere for 24 h at 37 °C in a 5% CO_2_ incubator. Following the protocol described in Section 4.4, the cells were treated with either cortisone alone or a combination of cortisone and CNF. After 24 h of treatment, the cell culture supernatants were collected, and the cortisol concentrations were measured using a cortisol ELISA kit (Beyotime Inc., Shanghai, China) according to the manufacturer’s instructions.

### 4.7. Triglyceride, Total Cholesterol and Fatty Acid Quantification Assays

SZ95 cells were seeded into 6-well plates at a density of 1.5 × 10^5^ cells/mL (2 mL per well) and treated with either cortisone alone or a combination of cortisone and CNF, following the protocol described in Section 4.4. Cell pellets were collected by trypsin digestion followed by centrifugation. To extract cellular lipids, 500 μL of isopropanol was added to each pellet, and the samples were homogenized in an ice bath. The resulting mixture was centrifuged at 12,000 rpm for 5 min at 4 °C, and the supernatant was carefully collected for subsequent analysis. The concentrations of triglycerides, total cholesterol, and free fatty acids were determined using the corresponding quantitative assay kits (Beyotime Inc., Shanghai, China) according to the manufacturer’s instructions. Standard curves were constructed using triglyceride, cholesterol, and palmitic acid standard solutions, respectively.

### 4.8. Total RNA Extraction and RT-qPCR Detection

Total RNA was extracted using TRIzol^®^ reagent (Thermo Fisher Scientific, Waltham, MA, USA) following the protocol described in Section 4.2. Cells were seeded in 6-well plates at 3.0 × 10^5^ cells/mL, cultured for 24 h, and then treated with cortisone alone or in combination with CNF for an additional 24 h. RNA was extracted by chloroform–isopropanol precipitation, dissolved in RNase-free water, and diluted to 200 ng/μL. cDNA was synthesized using a reverse transcription kit according to the manufacturer’s protocol [35]. Relative gene expression was quantified via the 2^−ΔΔCT^ method with ACTIN as the internal control. Primer sequences are listed in Table 1.

### 4.9. Western Blotting

Following the treatment protocols described in Section 4.4, the culture medium was aspirated, and total cellular proteins were extracted using RIPA lysis buffer. Protein concentrations were determined using the BCA Protein Assay Kit. Equal amounts of protein were separated by SDS-PAGE and transferred onto 0.2 μm polyvinylidene difluoride (PVDF) membranes. The membranes were blocked with 5% non-fat milk or 1% bovine serum albumin (BSA) for 2 h at room temperature and then incubated overnight at 4 °C with primary antibodies against Akt, p-Akt, mTOR, p-mTOR, PI3K, p-PI3K, SREBP-1, PPARγ, LXRα, C/EBP-α, PCNA, Bcl-2, and Bax (all diluted at 1:1000; Sanying Biotechnology, Wuhan, China). After extensive washing with TBST, the membranes were incubated with horseradish peroxidase (HRP)-conjugated secondary antibodies (1:1000) for 1 h at room temperature. Immunoreactive protein bands were visualized using an enhanced chemiluminescence (ECL) kit (Beyotime Biotechnology) and captured with the ChemiDoc XRS+ imaging system. Band intensities were normalized to β-actin, which served as the internal loading control [36].

### 4.10. Data Processing and Statistical Analysis

The data analysis was carried out using GraphPad 11, and data were expressed as mean ± standard deviation. The comparison between groups was assessed through analysis of variance (ANOVA) followed by Tukey’s test. A *p*-value less than 0.05 was considered to be statistically significant.

## Figures and Tables

**Table 1:** Primer design for SZ95.

Primer Name	Primer Sequence
*DGAT*	F: 5′-CCCATGCCTGGTTATTGCG-3′
	R: 5′-GGAGCCCACTGGAGTGATAGAC-3′
*FAS*	F: 5′-CATCTGGACCCTCCTACCTCTG-3′
	R: 5′-CTGTGTACTCCTTCCCTTCTTGG-3′
*ACC*	F: 5′-GCACAATCCTTAGGGACAACATAC-3′
	R: 5′-ATGCCAATCTCATTTCCTCCTG-3′
*ACTIN*	F: 5′-CACCCAGCACAATGAAGATCAAGAT-3′
	R: 5′-CCAGTTTTTAAATCCTGAGTCAAGC-3′

## Data Availability

All data generated or analysed during this study are included in this published article.
